# Clinician Well-Being: Addressing Global Needs for Improvements in the Health Care Field: A Joint Statement from the American College of Cardiology, American Heart Association, European Society of Cardiology, and World Heart Federation

**DOI:** 10.5334/gh.1067

**Published:** 2021-07-13

**Authors:** Laxmi S. Mehta, Mitchell S. V. Elkind, Stephan Achenbach, Fausto J. Pinto, Athena Poppas

**Affiliations:** 1 Division of Cardiology, Department of Internal Medicine, The Ohio State University Wexner Medical Center, Columbus, Ohio, US; 2 Department of Neurology, Vagelos College of Physicians and Surgeons, and Department of Epidemiology, Mailman School of Public Health, Columbia University, New York, New York, US; 3 Department of Cardiology, Friedrich-Alexander University Erlangen-Nürnberg, Erlangen, DE; 4 Department of Cardiology, Santa Maria University Hospital, CHULN E.P.E., Lisbon, PT; 5 Division of Cardiology, Department of Internal Medicine, Warren Alpert Medical School of Brown University, Providence, Rhode Island, US

**Keywords:** burnout, practice efficiency, professional fulfillment, resiliency, well-being

*“Health is a state of complete physical, mental, and social well-being and not merely the absence of disease or infirmity.”*

The World Health Organization [1]

As clinicians, we strive for improved health for our patients; yet, it is increasingly clear that our own well-being is an essential component of the quadruple aim: to improve population health, enhance patient experience, reduce costs, and improve the work life of health care workers [2]. Clinician well-being is described as experiencing satisfaction and engagement with work, while also having a feeling of professional fulfillment and a sense of meaning in work [3]. Conversely, burnout is an occupational phenomenon that is defined as emotional exhaustion, depersonalization, and a sense of low personal accomplishment in a perceived stressful work environment [4]. Although burnout is a sign of clinical distress and a barrier to clinician well-being, its absence alone does not confer a state of well-being. Rather, burnout is one of the more extreme negative components along the spectrum of clinician well-being (Figure 1), and can coexist with other common mental conditions (e.g., anxiety and depression).

**Figure 1:**
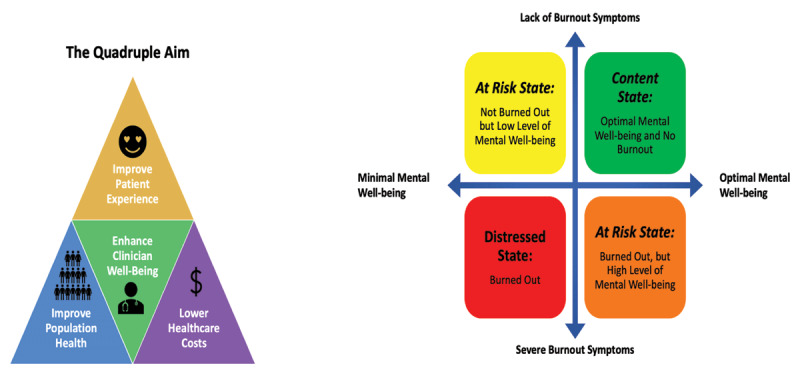
Well-Being in Cardiology. The quadruple aim **(left)** and the spectrum of burnout symptoms and mental well-being **(right)**.

## Drivers of Burnout and its Consequences

**Figure 2:**
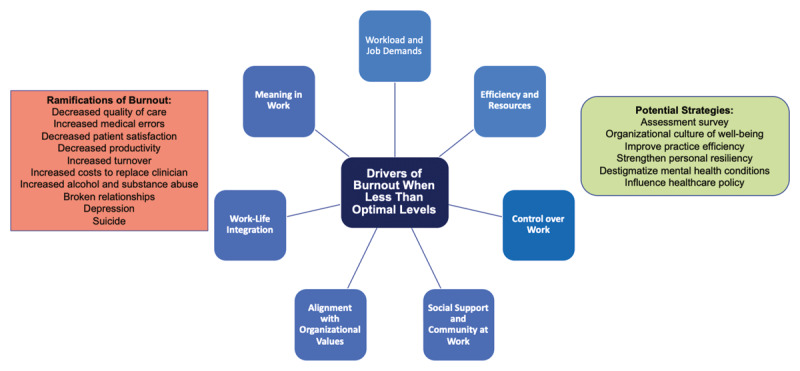
The Personal and Professional Ramifications and Drivers of Burnout and Potential Strategies to Address Clinician Well-Being. The personal and professional ramifications of burnout **(left)**, drivers of burnout **(center circle)** when less than optimal levels, and potential strategies to address clinician well-being **(right)**.

Over the past several decades, there have been dramatic changes in health care with the evolution of technology, increased regulatory burden, and the exponential growth of clerical burden, partly brought about by the widespread introduction of electronic health records. These developments come at a cost to the well-being and work-life balance of clinicians. There are several key drivers (Figure 2) that can influence clinician burnout when the levels are less than optimal: workload and job demands, efficiency and resources, control over work, work-life integration (WLI), alignment of individual and organizational values, social support/community at work, and meaning in work [3,5]. Furthermore, women are under-represented in cardiology and may have more stressors contributing to burnout due to lack of career promotion, inequalities in resources (financial), and disparities in mentorship while working in environments that lack diversity, equity, inclusion, and belonging [6].

Unfortunately, there are serious personal and professional costs associated with clinician burnout (Figure 2). Personal ramifications of burnout include higher rates of alcohol abuse, substance use, dysfunctional relationships, depression, and suicide [7,8,9,10]. Although mental health conditions can occur along the spectrum of burnout, it is important to recognize that not all burned out clinicians will develop mental health conditions, and not all clinicians with mental health conditions will experience burnout. Professional ramifications of clinician burnout include higher rates of medical errors, lower quality of care, decreased patient satisfaction, increased disruptive behavior, and loss of professionalism accompanied by a decreased level of empathy [3,5,11,12,13,14]. Health system expenditures related to the burnout of staff are steep when taking into account institutional costs due to decreased productivity and increased clinician turnover [15,16,17,18]. In the past year, the coronavirus disease 2019 pandemic has caused additional strain on clinicians through increased patient mortality, personal and family safety concerns, fear of the unknown, and increased work demands. Although clinician well-being is now openly discussed in the media, the stigma of seeking mental health counseling among clinicians remains.

Assessing burnout and its drivers by conducting surveys is a critical step to understanding the factors that need to be addressed to cultivate an engaged and efficient workforce and to improve clinician burnout. Hence, burnout is a metric to measure and monitor, whereas clinician well-being is the goal in cardiology and medicine. With this joint statement, the American College of Cardiology (ACC), American Heart Association (AHA), European Society of Cardiology (ESC), and World Heart Federation call for global action in health care reform, research, and policy development to address clinician well-being. Further, we aim to generate awareness about the impact of burnout and potential solutions to improve clinician well-being in cardiovascular medicine.

## Cardiology Burnout Data

Survey data among 2,274 U.S. cardiologists and fellows-in-training reported that more than one-quarter reported being burned out and almost 50% were stressed; only 23.7% reported that they were enjoying their work. Women reported burnout more frequently compared with men. Also, midcareer cardiologists more frequently reported burnout compared with fellows-in-training and early- or late-career cardiologists. Neither type of practice setting nor type of cardiovascular subspeciality had an impact on burnout, but burned-out respondents did report more time spent in direct clinical practice. Overall, most were satisfied with their career; however, burned-out respondents were less satisfied with achieving their professional goals or their desired financial compensation, and were less likely to recommend cardiology as a career. Compared with their peers, burned-out physicians were less likely to report feeling valued or being treated fairly at work. Drivers associated with burnout among cardiologists include lack of control over workload, a hectic work environment, misalignment of values, and insufficient documentation time [19]. Data among U.S. physicians from all different specialties demonstrated that 39.8% reported burnout and 42.7% satisfaction with WLI, whereas other data among physician assistants and nurses have shown lower burnout rates (35.8% and 29.7%, respectively) and higher WLI rates (65.3% and 55.5%, respectively) [20,21,22]. There is a dearth of survey data specific to cardiovascular professionals; however, the drivers of burnout among cardiologists may likely be extrapolated to the cardiovascular workforce.

## Health Care Organizational Strategies

Health care organizations have predominately focused on the concept of “fixing the employee” with individual-focused programs (self-resiliency and stress management training) as the solution to improving well-being; however, much more effort needs to be tailored to systemic issues that affect the work environment. Intentional refinement of practice environments with highly functioning teams within which clinicians can optimally care for patients enables all team members to find value and purpose in their work and can result in improved outcomes for the health care organization and patients. It is imperative for health care organizations to support the psychosocial health of their employees and be accountable for a holistic approach. One such method is the Stanford WellMD Professional Fulfillment Model, which incorporates culture of wellness, practice efficiency, and personal resiliency domains [23], while also taking into account the intrinsic (e.g., recognition, trust, meaning in work) and extrinsic (e.g., call schedule, compensation, technology) driving factors that can be modified.

Organizational infrastructure is necessary to provide the foundational means to maintain work environments within which clinicians may thrive. The burnout and wellness hierarchy model, adapted from Maslow’s hierarchy of needs, provides a prioritized stepwise approach for health care leaders to address clinicians’ professional fulfillment: basic physiological and mental health needs first; safety and security second; respect from administrators, colleagues, and patients third; appreciation and interpersonal connection fourth; and finally, resources and time to treat patients and practice medicine fully [24]. For instance, some techniques by which health care organizations could foster a healthy and resilient workforce include ensuring adequate rest and recovery time, supplying appropriate personal protective equipment, reducing systemic inefficiencies and improving team dynamics, compensating fairly and transparently, and facilitating more direct time with patients.

In addition, health care organizations need to provide employees with a structure to allow for confidential reporting of mistreatment and also to destigmatize clinicians’ access to mental health resources. Hospital credentialing committees need to refine their inquiries into mental health conditions so that it does not perpetuate a culture of either under-reporting of mental health conditions or undertreatment due to the fear of the loss of hospital privileges among clinicians.

Regular assessment of clinician burnout is essential to understand the effectiveness of implemented strategic pilot projects. Similar to the concepts of prevention of cardiovascular disease, secondary prevention is achieved with targeted approaches to prevent recurrent burnout; however, investment in primary and primordial prevention is equally crucial to reduce or potentially avoid burnout all together.

## Role of Medical Specialty Societies

Clinician wellness is a broad entity that requires medical specialty societies to develop a multipronged approach to make meaningful progress. To support their members, medical societies will need to continue to provide recommendations to health care organizations and advocate for meaningful health policy changes. Furthermore, development of cardiology-specific tools that may improve practice efficiency or clinician knowledge base in a timely and convenient fashion is imperative. Medical specialty societies are also key in developing a sense of community for their members. Engaged members of medical specialty societies often cite global social networks as a recurring theme for ongoing involvement in societal committees and attendance at national conferences. However, medical societies will need to continue to expand their initiatives in diversity and inclusion so that all members feel valued and have a sense of belonging. In addition, the medical specialty societies will need to consider innovative ways to deliver educational content and offer networking opportunities, especially in light of resource constraints and increased need for virtual platforms.

In March 2020, the ACC’s Board of Trustees commissioned the Task Force on Clinician Well-Being to help focus its strategy to address clinician well-being. Several surveys have been launched to assess the prevalence of and drivers of burnout amongst ACC members. Member sections are integrating well-being themes in their various offerings to members, including toolkits, webinars, and conferences. Recent webinars share cardiologists’ perspectives of mental health conditions as well as inform the viewers regarding signs and symptoms of anxiety and depression, encourage treatment, and provide members the contact information for confidential counseling resources. Additionally, clinician well-being was incorporated into the 2020 ACC/AHA Professionalism and Ethics conference, and the formal recommendations were recently published [25].

Also, in Europe, nearly 4,000 medical faculty and researchers from 17 ESC member countries in 2018 completed the ESC-sponsored “Culture and Leadership in Cardiology in Europe” C-Change Faculty Survey. Overall, cardiologists and research professionals have high leadership aspirations and a high degree of personal satisfaction in their work. However, approximately one-quarter of individuals did not view their institutions as providing the best opportunities for meaningful work, and one-third did not feel they were supportive of career advancement. The ESC has been using these findings to guide future strategic initiatives that aim to provide appropriate support for cardiologists in Europe and enable satisfaction within their work environment.

The ACC, AHA, ESC, and World Heart Federation acknowledge that clinician well-being is paramount to providing high-quality patient care and are committed to improving the well-being of the cardiovascular workforce.

Medical specialty societies will need to continue to identify and promote for the reduction of administrative complexities and practice inefficiencies, which are burdensome and hinder professional fulfillment and patient care. Advocating for appropriate initiatives to improve clinician well-being and mental health resources, while also influencing the elimination of non-essential regulation and document requirements, is key. Furthermore, assisting in combatting the stigma of mental health conditions and the negative ramifications by licensing boards of a history of treated medical health conditions are crucial endeavors for medical specialty societies. Collaboration with other specialties is necessary, including leveraging the expertise of psychiatrists and clinical psychologists. The House of Cardiology will continue to work together and strategize to maintain the well-being of our profession and keep the joy in medicine.
